# Potential Uses of Musaceae Wastes: Case of Application in the Development of Bio-Based Composites

**DOI:** 10.3390/polym13111844

**Published:** 2021-06-02

**Authors:** Juan Pablo Castañeda Niño, José Herminsul Mina Hernandez, Alex Valadez González

**Affiliations:** 1 Grupo Materiales Compuestos, Universidad del Valle, Calle 13 No. 100-00, Cali 76001, Colombia; jpagroin@gmail.com; 2 Unidad de Materiales, Centro de Investigación Científica de Yucatán, A.C., Calle 43 #. No. 130, Col. Chuburná de Hidalgo, Mérida, Yucatán 97205, Mexico; avaladez@cicy.mx

**Keywords:** musaceae, lignocellulosic wastes, natural fibres, bio-based composites, thermoplastic starch

## Abstract

The Musaceae family has significant potential as a source of lignocellulosic fibres and starch from the plant’s bunches and pseudostems. These materials, which have traditionally been considered waste, can be used to produce fully bio-based composites to replace petroleum-derived synthetic plastics in some sectors such as packaging, the automotive industry, and implants. The fibres extracted from Musaceae have mechanical, thermal, and physicochemical properties that allow them to compete with other natural fibres such as sisal, henequen, fique, and jute, among others, which are currently used in the preparation of bio-based composites. Despite the potential use of Musaceae residues, there are currently not many records related to bio-based composites’ developments using starches, flours, and lignocellulosic fibres from banana and plantain pseudostems. In this sense, the present study focusses on the description of the Musaceae components and the review of experimental reports where both lignocellulosic fibre from banana pseudostem and flour and starch are used with different biodegradable and non-biodegradable matrices, specifying the types of surface modification, the processing techniques used, and the applications achieved.

## 1. Introduction

The current period lived by humanity is related to the plastic age due to its dependence on developing diverse low-weight, durable, and low-cost products, generating a world production of approximately 260 million tons each year. However, the massive consumption of plastic products and their rapid disposal has contributed to the accumulation of waste in remote parts of the planet, including landfills, land surfaces, and ocean waters [1]. In ocean waters, plastic waste is released from the continents through mouth basins and is concentrated by marine currents in specific locations in the North Atlantic and Pacific Oceans (North and South). This material exposed to marine conditions with solar exposure contributes to the generation of microplastics through photodegradation and fragmentation, being consumed by a wide variety of aquatic living beings. The ecosystem’s impact is also evidenced by various species death that ingests plastic macroparticles [2]. The single-use containers and packaging sector contributes with different plastic items (such as glasses, plates, containers, cutlery, and straw), with close to 50% of the total plastic discarded in the mentioned areas, being two-thirds constituted by thermoplastic resins. The alternatives to counteract the environmental problem caused by plastics are the use of biodegradable polymers, recycling, and/or the use of synthetic polymers as fuel [1,3]. Using biodegradable materials such as starches, lignocellulosic fibres, polylactic acid, polyhydroxyalkanoates, and environmentally friendly bio-based composites can be developed, providing properties that compete with traditional non-biodegradable composites or materials. Among these, starch and lignocellulosic fibres blends stand out due to their high availability, and they provide a high modulus of elasticity, lower water absorption, greater thermal stability, mitigation of the starch retrogradation effect, and reduction of costs in bioplastics that currently demand the food packaging sector [4]. These macromolecules can be found in various crops such as cereals, tubers, roots, and fruits [5,6]. Among the different fruits, the banana stands out for being a source of raw material for the development of new bio-based composites, highlighting that the matrix can be obtained from the starch and flour coming from the bunch, while the reinforcement is extracted from the lignocellulosic fibres coming from the pseudostem [7]. For the separation of banana’s and plantain’s starch and flour, physical and chemical processes are carried out, which require gelatinisation of the material through temperatures above 70 °C and mechanical action to obtain its respective thermoplastic state [8].

In contrast, the lignocellulosic fibres must be extracted by mechanical methods and subsequently subjected to a surface modification of a physical and/or chemical type to achieve greater interfacial adhesion with the matrix, achieving the expected benefits in the final bio-based composite [9]. Various transformation techniques such as compression moulding, extrusion, and injection moulding can be used to obtain composites with different mechanical, thermal, and physicochemical properties, allowing them to be implemented in different social sectors. Following the above, in this study, a review was carried out of research related to the generalities, methods of extraction, and adaptation of the Musaceae family, starches, flours, and lignocellulosic fibres of the Musaceae pseudostem, development of bio-based composites, including their mechanical, thermal, and physicochemical characterisation, as well as their applications in different sectors of society.

## 2. Musaceaes

Bananas and plantains are found in this genus, locating their cultivation in the extension belonging to the tropical and subtropical regions. Their crops are perennial with a high growth capacity than other fruit trees while producing fruit or bunches throughout the year. Due to their nutritional value, bananas and plantains are the fourth most consumed crop worldwide, after rice, wheat, and corn. These Musaceae represent an essential source of income for several rural families who work directly or indirectly [7].

### 2.1. Origin and Initial Distribution

Bananas and plantains originated in Southeast Asia and the Western Pacific regions from their diploid ancestors’ development and are characteristic of being inedible and seed-bearing [10,11]. These edible Musaceae originated from the natural crossing of two wild species, the *Musa acuminata Colla* and the *Musa balbisiana Colla*, containing genomes A and B, respectively, being the genomic contributors to the generation of hybrids and polyploids. Several of these hybrids are parthenocarpic, presenting a sterile and triploid female genomic structure, edible fruit, and their propagation is asexual through the use of shoots [7,12]. The triploid Musaceae initially located in South East Asia (India and the Philippines) were subjected to selection according to their vigour, size of the fruit, and their capacity to adapt, being superior characteristics concerning their diploid ancestors. The first records of edible Musaceae come from India and are 2500 years old. In addition, it has been identified that the genes of *M. acuminata* contribute to the resistance of hybrids to drought and provide a sweet taste characteristic of bananas, while the genes of *M. balbisiana* contribute to increased resistance to disease and nutritional value, finding higher starch contents and allowing cooking, which is characteristic of plantains [7,8,13]. The distribution of bananas and plantains outside Asia consisted of the transport of sprouts or corms (asexual propagation) to different places in the tropical region, initially passing to Madagascar in 500 AD; later, it entered the eastern coasts of Africa, and the crops expanded through the Congo until they reached the western coasts of the continent between the 14th and 15th centuries. Through the Portuguese colonisations, the Musaceae continued their expansion in the Canary Islands; after the conquest of the Canary Islands by the Spanish and the colonisation of Santo Domingo (Dominican Republic), the Musaceae reached America in 1516 (16th century), and finally, they reached the coasts of Central America and the Caribbean [7,14]. These herbaceous monocotyledonous plants arrived in Colombia due to the Spanish’s arrival in the 16th century [15,16].

### 2.2. Taxonomic Classification

According to Charles Linnaeus, in 1783, he gave the name *Musa sapientium* to all the bananas that can provide a sweet taste when ripe and are consumed fresh, while he called the plantains as *Musa paradisiaca*, since they must be cooked for consumption from their green state (presence of starch) until they are ripe. However, the respective names are apparent species and not real species, as bananas and plantains are related to the AAB group’s triploid hybrids [7,14]. Due to the above, the classification of greater specificity has been required to differentiate the Musaceae’s characteristics.

### 2.3. Modern Classification of Musaceae

Based on the genomic contributions of *M. acuminata* (genome A) and *M. balbisiana* (genome B), between 200 and 300 hybrids have originated, finding the participation of triploid Musaceae (33 chromosomes) greater than half of all hybrids existing. In contrast, tetraploids (44 chromosomes) are found in a low proportion. Other wild species contribute different genomes, such as the *Musa schizocarpa* (S genome) and the *Musa textilis* (T genome); however, these do not contribute to hybrids for food purposes. The classification of bananas and plantains are obtained from the scoring technique according to the 15 characters of the Musaceae (see Table 1), using a scale of scores between 15 and 75, with the lowest score representing pure *M. acuminata*, while the highest score represents pure *M. balbisiana*. The intermediate scores relate the different contributions of the native species and the ploidy level in each interspecific hybrid [7,11,14]. Table 1 lists some Musaceae species in each genomic group, differentiating between sweet bananas, plantains, and cooking bananas [7,10]. The largest plantain producers worldwide are (1) Cameroon, (2) Ghana, (3) Uganda, and (4) Colombia [17]. In the case of bananas, the primary producers are (1) India, (2) China, (3) Indonesia, (4) Brazil, (5) Ecuador, (6) the Philippines, (7) Angola, (8) Guatemala, and (9) Colombia [18]. In Colombia, the plantain species of greater use for cultivation are Dominico-Hartón, Dominico, and Hartón from the use of 405,910 hectares in the planted area, generating a production of 3,534,083 tons and a production yield of 8.71 tons/hectare [15,16], while the Banana, Cavendish, and Gros Michel species are harvested from the use of 82,905 hectares, producing 2,052,991 tons and a yield of 24.75 tons/hectare in the year 2015 [15,16,19,20].

### 2.4. Morphological Characteristics and Development of Musaceae

The species that make up this family are described as monocotyledonous, herbaceous (does not contain a wood structure), and have an evergreen leaf due to the generation of shoots or corms to replace the dead leaves from the mother plant [7].

#### 2.4.1. Root System

The plant is planted vegetatively or asexually, identifying a fleshy root from the beginning of the shoot or corm development when planted. The root is classified as primary, secondary, and tertiary. The primary roots have a diameter of between 5 and 8 mm, are white when healthy, and generate between 200 and 1000 units in the shoot or corm. Secondary and tertiary roots develop in each primary root, which are characterised by having smaller diameters and lengths than the primary roots. Their function consists of absorbing water and minerals, their adequate development being essential to take advantage of the plant’s potential [7,26]. When comparing bananas and plantains, Robinson and Galán [7] reports that the proportion of secondary and tertiary roots in the banana is 22 and 77%, while in the plantain, it is 53 and 46%, respectively. These characteristics explain the lower productivity and yield of bunches produced by the plantain.

#### 2.4.2. Rhizome and Sprouts

The shoots can also be called tuberous rhizomes, corms, or bulbs and are generated adjacent to the mother plant. Their diameter can reach values between 200 and 300 mm, and their height varies according to the individual vigour. The shoot structure is made up of external internodes covered by leaves, while the inside is a central cylinder made up of a fine tissue called parenchyma, which is characteristic for having starch and is covered by a bark with a thickness of 10 to 30 mm. The formation of each shoot begins when the mother plant begins to generate its leaves. It has 45% dry matter; however, when the mother plant generates fruit with its degree of ripeness for the harvest, the dry matter content is reduced to 30%, as it serves as a reserve to contribute to the development of the bunch. Each mother plant can produce up to 15 shoots [7].

#### 2.4.3. Leaves

When the shoot is planted, its foliar development comes from the pseudostem’s center and requires 6 months to complete the foliar stage until the development of the largest leaves is achieved, being essential to have between 10 and 15 leaves that cover a foliar area of 25 m^2^, and then the flowering is generated [7,14]. Gowen [14] reports that the individual leaf area of banana trees is between 1.27 and 2.80 m^2^, while in plantains, it is between 0.68 and 0.92 m^2^.

#### 2.4.4. Pseudostem

In the leaves’ development, the sheaths are piled up and increase their thickness to form the pseudostem, and the generation of new leaves is maintained until the maximum height is reached, with values between 2 and 8 m [14]. A mature pseudostem must have a robust and fleshy structure, which is divided into four stages, participating with different volumes: (a) external (≈41%); (b) intermediate (≈27%); (c) internal (≈21%); and (d) shell (11%) [27]. This allows it to support a bunch weighing more than 50 kg and its water content is between 90 and 95%, the lignocellulose fibre content is between 1.6 and 8% on a wet basis, and the remaining difference corresponds to the parenchyma, with a value of up to 4% [7,28]. However, Aziz et al. [29] report a lignocellulose fibre content of 29.9% on a dry basis. Another macromolecule found in this part of the plant, specifically in the shell, is starch [27,30].

#### 2.4.5. Inflorescence

During the plant’s development, its apical growth (pseudostem and leaves) ends, and the inflorescence begins, due to a possible hormonal induction, synthesising gibberellic acid in the pseudostem after generating a certain number of leaves [14]. Female and male flower bracts are formed, enclosing themselves in axillary meristematic cushions to form the flowers. The flowers are organised in nodal clusters on a peduncle to form the inflorescence, with the male flowers at the end of the peduncle, forming a conical structure called the bell or rachis. Meanwhile, in the basal nodes, the female flowers are found, which are organised into 5 to 18 bouquets [7].

#### 2.4.6. Bunch

The peduncle increases in length and diameter, while the female flower bracts tend to expand with a subsequent upward reorientation after a few weeks from the start of flowering and end with each bract increasing in diameter until the fingers of a banana or plantain bunch are ready for harvest. In the same way, the proportions of the components of each finger are modified, generating an increase in the volume participating in the pulp, and the proportion belonging to the peel is reduced until an optimum size of each finger of the bunch is achieved in a time between 85 and 110 days after the start of the inflorescence. Bunch size depends on the genomic group, the crop cycle, temperature, plant vigour, and operator management. In each bunch, 16 or more hands can be obtained, while each hand can contain 10 and 30 fingers. A bunch of bananas can vary between 15 and 70 kg, while plantains can weigh between 7.5 and 14 kg [7].

#### 2.4.7. Fruit’s Development and Ripening

Each finger belonging to the bunch of a Musaceae, during its dependence on the plant, generates starch in its pulp and increases its physical dimensions (increase in diameter until the bunch is harvested). After the bunch is harvested, the climatic stages contributing to its ripening begin.

- The pre-climateric stage, first stage, or green stage begins after the bunch harvest until some physical change is generated, which is characteristic of climatic breathing. There is a slow metabolic activity, and the commercial objective is to prolong it through storage at a temperature of 13 °C and/or the use of controlled atmospheres. The harvest of a Gros Michel banana, Dominico Hartón, and FHIA 20 plantains requires a time between 8 and 10 weeks after the inflorescence in the department of Caldas (Colombia). Chávez-Salazar et al. [10] reported the respective contents of starch being 5.78, 12.73, and 10.18% in the humid base and 18.73, 32.22, and 31.67% in the dry base, respectively, evidencing a higher content in plantains. On the other hand, its °brix did not exceed 11, being characteristic of a green and physiologically immature plantain during 9 days of storage. In a second study, the increase of starch presented in a Dominico Hartón plantain’s pulp was determined by comparing the harvest between weeks 14 and 18 after inflorescence, reporting 56.5 and 74.8% on a dry basis, respectively [31,32]. In another study conducted in Malaysia, starch from green bananas can be obtained between 70 and 80% on a dry basis [33]. The peel also generates a starch contribution between 16.6 and 48.5% in the dry base [34].
- The climatic stage. Ethylene is produced in the second stage, being a climacteric respiration product, through two enzymes, 1-aminocyclopropane-L-carboxylic acid synthetase and ethylene-forming enzyme. This respiration is based on the rapid absorption of oxygen and carbon dioxide generation [7,35].
- Maturation stage. Various changes are generated in the fruit such as the peel’s change of colour, going from dark green, followed by light green, and ending in yellow. In addition, there is evidence of softening of the skin and pulp, converting starch into reducing sugars, and generating aroma [7,14]. Barrera et al. [36] reported an increase in total solids and reduction of the rigidity and pH in the fruit as the ripening time increases due to the degradation of the starch, generating an accumulation of reducing sugars, mainly glucose, fructose, and sucrose, until a content of 35–40% is achieved in the fruit when it has an intense yellow peel.
- Final stage. At the end of the breathing process, the fruit’s physiological death is obtained, revealing a brown to black skin, and the pulp changes colour, going from white to brown with a gelatinous texture [7]. The time required for the ripening mentioned in the above stages is between 13 and 20 days [7,14].

### 2.5. General Climatic Requirements

Among the conditions necessary for the development of the crop, an altitude from sea level to 2000 m.a.s.l. is required, the optimal temperature in the crop should be between 26 and 27 °C, rainfall between 120 and 150 mm per month or 1800 mm per year, wind speed should not exceed 20 km/hour to avoid physical damage, and the type of soil should have a topography between flat to undulating, deep, well-drained, fertile, and with a good amount of organic matter [14,37].

## 3. Starchy Products Obtained from Banana and Plantain Bunches

Starches and flours for processing in the food and non-food sectors can be obtained from the Musaceaes’ bunches. The most relevant physicochemical, physical, and thermal properties for processing and extraction or isolation methods are reported below.

### 3.1. Native Starch

Starch is the carbohydrate that occurs in the greatest proportion in the endosperm of cereals, reserve parenchyma in tubers, roots, and fruits, and is found in the form of granules in the cells, forming discrete structures. Their size can vary between 1 and 100 µm, depending on their botanical source, although there is an inevitable heterogeneity of size in the same plant. The shape can vary, being round, elliptical, oval, lenticular, or polygonal. According to the above, the smallest granules correspond to rice and amaranth starches, with diameters between 1 and 10 µm. Potato starches have diameters between 20 and 100 µm, while those of maize starches are between 15 and 20 µm, and those of cassava starches are between 5 and 45 µm [5,38,39,40]. In general, the different starch granules’ surface area varies according to the botanical origin and is a question mark in several studies. Starch is a biopolymer composed of anhydrous α-linked glucose units. This biomolecule is made up of two polysaccharides, amylose, formed by α−1,4 bonds, and its structure is based on linear chains and amylopectin, presenting α−1,4 and α−1,6 bonds, the latter bonds being responsible for generating branches that give starch its native semi-crystallinity [5,6]. The proportions and molecular weights of amylose and amylopectin in starch from any botanical source determine the physicochemical properties. The molecular weight of amylose chains is in the order of 1 to 2 × 10^5^ Daltons, and their ratio varies between 20 and 30%. In amylopectin, on the other hand, the molecular weight is greater than 2 × 10^7^ Daltons and ranges from 70 to 80%. Both polymers are made up of alpha-D-glucose units. Amylopectin consists of 20 to 30 glucose units [41]. The physicochemical properties usually studied in starch are proximal composition, colour analysis, molecular weight, solubility, swelling power, water absorption, and relative amylose content. Structural characterisation (size, shape, and crystalline nature of the granule) and rheological characterisation (swelling power, gelatinisation temperature, and viscosity in solutions) are also taken into account, these properties being complementary in the physicochemical study [8,33,38,42,43].

### 3.2. Flour 

It is based on the grinding, sieving, and drying of a seed, tuber, root, or fruit, maintaining its original components (starch, fibre, fat, protein, and ash) and reducing its water content. Starch is one of the macromolecules with the most significant participation, achieving close to 60% [44].

### 3.3. Starch and Flour from Musaceae

**Figure 1:**
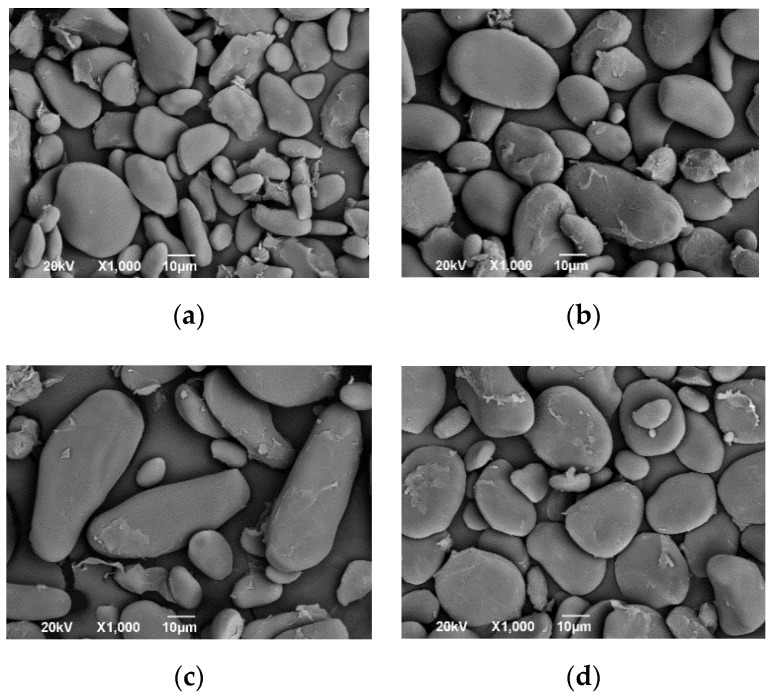
SEM micrograph of plantain starch granule of four varieties: (**a**) Gros Michel; (**b**) Dominico; (**c**) Cachaco; (**d**) Guineo.

The shape of the starch granules from the Musaceae tends to be elongated oval with some irregular endings (Figure 1). In the case of the Gros Michel banana starch granules, their dimensions are range from 10 to 20 µm wide and 35 to 70 µm long, while the starches of the Dominico hartón and FHIA 20 plantains range from 20 to 30 µm wide and 18 to 32 µm long, and 50 to 65 µm wide and 39 to 75 µm long, respectively. The amylose/amylopectin ratio was reported as 22.76/77.24 in Gros Michel, 31.12/68.88 in Dominico hartón, and 28.58/74.42 in FHIA 20 [10]. Since amylose is a polymer constituted by linked glucose, regular starches present values close to 20% [5,6], with Musaceae being a starch source with a relevant contribution of amylose. The size of the starch granule, the degree of crystallinity, the amylose/amylopectin ratio, and the interactions of the polymeric starch chains in the amorphous phase interfere in the conditions of starch processing where the temperature of gelatinisation is involved [45]. Due to their amylose content with values close to 30%, most of the banana and plantain species achieve gelatinisation temperatures from 70 °C and are part of the starches group resistant to enzymatic actions [8]. In the flour obtained from Musaceae, its composition includes protein contributions between 2.57 and 3.16%, lipids with values lower than 0.56, fibre between 1.18 and 1.65%, and starch between 83.2 and 88.3% [46,47]. When evaluating the Terra plantain, the particle distribution is slightly lower in the flour than the starch, finding an average value of 31.7 and 47.3 µm, respectively. In contrast, the gelatinisation temperature of the flour is higher concerning its starch, being 76.2 and 74.9 °C, respectively, due to the more significant presence of fibre and fat in the flour, giving greater thermal stability to the starch granules before gelatinisation [46].

### 3.4. Methods of Extracting Starch and Flour from Musaceae

Obtaining banana starch is based on wet milling from a blender or disc mill and the use of a solvent. Among the types of solvents, there are water, sodium bisulfite, citric acid, sodium hydroxide, or sodium sulfite. One or more solvents can be considered to extract starch to achieve higher yields and purity [48]. Starches from bananas and plantains have achieved a purity of between 82.3 and 99%, with a considered variation depending on the extraction method, degree of ripening, and the species to be used [10,48]. In the process of starch extraction corresponding to the banana and plantain, the following unitary operations are required: washing, peeling, cutting of the pulp, immersion of sodium bisulfite, wet milling using a solvent, sieving using different opening sizes, centrifugation of the solvent, drying of the starch paste, dry milling, sieving, and packing of the starch [10]. To reduce processing times, spray dryer can be used. However, it is more expensive to purchase. In the case of flour extraction, a smaller number of unit operations are required, starting with the washing of each whole finger of banana or plantain, immersion in a solution of potassium metabisulphite (1%), cutting into chips, drying by forced convection, dry milling by use of a blade mill, sifting and packaging of the flour [48]. Yields vary according to the species, achieving 50% (dry base) in the Terra plantain, while in banana species such as Alukehel and Monthan, values of 31.3 and 25.5% were achieved on a dry base, respectively [46], while the Dominico hartón plantain was 65.9% on a dry base (25.7% on a wet base) [47]. 

## 4. Lignocellulosic Fibres from Banana Pseudostem

Lignocellulosic fibres for the development of new materials can be obtained from the pseudostem of the Musaceae. The most essential physicochemical, physical, mechanical, and thermal properties for their processing and extraction or insulation methods are mentioned below.

### 4.1. Lignocellulosic Fibres

Natural fibres are subdivided according to their origins: vegetable, animal, and mineral. Fibres from vegetables are found in the stem, leaves, seeds, fruit, wood, straw, or bagasse of cereals and fodder. The chemical composition and the structure of fibres are complex, since their organisation is similar to a composite material. It is designed by nature, forming a rigid matrix that contains crystalline microfibrils of cellulose merged with lignin and/or hemicellulose. Most fibres of vegetable origin, except for cotton, are composed mainly of cellulose, hemicellulose, lignin, waxes, and some water-soluble components [49]. Some of the properties of industrial interest in lignocellulosic fibres are high modulus of elasticity and tensile strength, low density and cost, easy processing, and resistance to corrosion and fatigue. However, they have some disadvantages such as high hygroscopicity and anisotropy, low compatibility with resins or materials of different nature, limited thermal resistance (up to 230 °C), and variation in the diameter and its aspect ratio (length/diameter). It can be modified during its processing in extrusion and injection moulding [50,51,52,53].

#### 4.1.1. Cellulose

Alpha-cellulose is the macromolecule with the highest proportion in fibres of vegetable origin, presenting a linear structure based on D-glucose units’ repetition and joined from alpha-1,4 glycoside bonds with an average degree of polymerisation of 10,000. Each repeating unit contains three hydroxyl groups with the capacity to form hydrogen bridges and thus participate in its crystalline packaging and cellulose’s physical properties [52]. Cellulose has a semi-crystalline structure, consisting of amorphous and crystalline regions. The crystalline structure of natural cellulose is called cellulose I. It is resistant to strongly alkaline media and oxidising agents but is easily hydrolysed in acidic media to form water-soluble sugars. The crystallinity index given by cellulose in lignocellulosic fibres can reach maximum values of between 65 and 70%, using cotton, flax, and hemp as a reference [53]. Cellulose is organised into rigid microfibrils with a modulus of elasticity values of around 130 GPa, which are organised in a unidirectional way on the cell walls, generating a microfibrillar angle concerning the growth direction of the plant that contains them, with values of between 6 and 49° [52,54], with greater resistance to tension and modulus of elasticity in fibres that have a lower microfibrillar angle [52]. The microfibrillar angle value depends on the plant’s maturity, type of species, conditions for fibre extraction, and agro-climatic conditions of the supplying crop [55].

#### 4.1.2. Hemicellulose

It is a polysaccharide composed of a combination of five to six carbon sugars. The polymer chain is short (degree of polymerisation between 50 and 300) and has branches containing lateral groups to give it its amorphous nature. It forms a matrix with the cellulose microfibrils, has a high hydrophilic character, and is soluble in alkaline media and easily hydrolysed in acidic media [54].

#### 4.1.3. Lignin

It is a component that gives rigidity to the plants. It is established as a complex three-dimensional copolymer with aliphatic and aromatic substitutes with a high molecular weight. Its chemistry has not been established with much accuracy, but most of its functional groups and the molecular units that compose it have been identified. It has been characterised by its high carbon content and low hydrogen ratios. Hydroxyl, methoxyl, and carbonyl groups have been identified in its structure; it is believed that the structural unit of lignin corresponds to 4-hydroxy-3-methoxy phenyl propane. Its structure is amorphous and hydrophobic, corresponding to a thermoplastic polymer, exhibiting a glass transition temperature of 90 °C and a melting temperature of over 170 °C. It does not hydrolyse in an acidic medium but is soluble in an alkaline medium at high temperatures where it can be easily oxidised [54]. According to the conditions set out above, the lignocellulosic fibre can be modified by removing the lignin or hemicellulose.

### 4.2. Pseudostem’s Lignocellulosic Fibres

The chemical composition, physical properties, stress, thermal stability, and water absorption in the lignocellulosic fibres of the Musaceaes pseudostem are reported below and compared with other botanical sources.

#### 4.2.1. Chemical Composition

Starting from the pseudostem structure, the cellulose and hemicellulose content of the lignocellulosic fibres of the individual stems decreases from the outer layer to the shell [27,30]. The chemical composition of the lignocellulosic fibres of the pseudostem of most bananas and plantains is based on the presence of cellulose between 31.3 and 64%, hemicellulose between 10.2 and 19.0%, lignin between 5.0 and 19.1%, and extractives up to 5.2% being represented by waxes and pectins [9,30,56]. In terms of cellulose content, its value is lower than that of lignocellulosic fibres from flax stalks (64 to 71%), hemp (68 to 74%), abutilon (67 to 71%), pineapple leaf (up to 82%), and cotton (85 to 90%) [50,52,57,58], establishing itself as a fibre with an intermediate cellulose content, since none of the banana or plantain species reach values greater than or equal to 70% (see Table 2). The lignin content is intermediate, requiring physical or chemical pre-treatment to facilitate processing. The moisture absorption of the fibres is between 9 and 10.9% in controlled conditions of relative humidity, temperature, and storage, being higher than that reported for cotton with 8.5% and ramie with 8.3%, while other sources such as flax present values (9.9%) within the range reported for plantain’s pseudostem fibres, and the fibres with the highest degree of water absorption are pineapple leaf fibres with 13%, which is followed by wool and abaca with 15% [30,50,56].

#### 4.2.2. Physical Properties

In terms of physical properties, the plantain pseudostem’s fibre diameter varies between 50 and 250 µm, while in bananas, it varies between 56 and 350 µm (see Table 3). Under the above, an important variation in the fibres’ diameter is found because the Musaceae’s pseudostem is constituted by four stages, finding three of them established by sheaths. In the case of the Nendran plantain, the external zone of the pseudostem, being made up of the first four sheath, presents 32.5% of fibres with diameters close to 250 µm, while the fibres with diameters close to 200, 150, 100, and 50 µm participated in 42.5, 17.5, 6.5 and 1%, respectively. Moving on to the next intermediate pseudostem’s stage, made up of sheaths numbered 5 to 9, a variation in the distribution of the diameters is reported, with the group of fibres with diameters of 250 µm being reduced to 16.5%, while fibres with diameters of 200 µm go on to 33.5% and fibres with diameters of 150, 100, and 50 µm increase to 36.5, 10.0, and 3.5%, respectively. Finally, in the internal zone, which is formed from sheath number 10 onwards, the fibres with the largest diameter show values close to 200 µm with a participation of 6%; the proportion of fibres with a diameter of 150 µm are reduced to 27.5%, while fibres with diameters of 100 and 50 µm increase their participation to 44% and 22.5%, respectively [59]. From the previous research, the highest concentration of lignocellulosic fibres with the largest diameter is located in the sheaths closest to the external surface of the pseudostem to provide greater resistance to the support of the weight of the bunch and aggressive external conditions. Some sources such as cotton and abutilon have smaller diameters than reported for musaceae. The density of the fibres of the Musaceae pseudostem is between 650 and 1500 kg/m^3^, which is within the characteristic values of lignocellulosic fibres and is lower than other types of fibre such as glass, which has a higher density of 2550 kg/m^3^, being a disadvantage for the latter when it is used as reinforcement in the manufacture of composites because it contributes to the increase in weight [9,30,52,55,60]. While the microfibrillar angle is between 10 and 15°, the microfibrils’ location in the fibres allows for greater resistance to tension [30].

#### 4.2.3. Tensile Mechanical Properties

Based on the chemical composition and physical properties, the pseudostem’s lignocellulosic fibres have a modulus of elasticity between 7.7 and 32 GPa, maximum tensile strength between 54 and 754 MPa, and a maximum elongation of 10.3%, with tensile properties with intermediate values concerning those identified in other plant sources [9,30,55], finding only flax fibres (≈1500 MPa), pineapple leaf (≈1627 MPa), and cotton (≈800 MPa) with greater tensile strength, while in its modulus of elasticity, it has a lower value than flax (27.6 GPa), jute (26.5 GPa), ramie (128 GPa), sisal (22 GPa), and pineapple leaf (82.5 GPa) [52]. Taking into account the structure of the Musaceae pseudostem, which is made up of three layers and a shell, we find a considerable distribution of diameters in the lignocellulosic fibres, increasing the deviation in the values of tensile strength and modulus of elasticity, highlighting the lower mechanical values in the fibres located in the sheaths that make up the inner layer of the pseudostem [30]. According to Table 4, as cotton provides the fibre with the most significant cellulose contribution, its greater resistance to tension is close to that shown by several authors who evaluate the fibre of the pseudostem from Musaceae. However, the minimum tension value of cotton fibres is higher than that reported by Musaceae. In the case of the coconut fibres, their tension and stiffness properties are lower than those of Musaceae fibres due to their high lignin content and microfibrillar angle (30 to 49°) [52]. The tensile and stiffness properties of fibres belonging to the Musaceae are not preserved and are considerably reduced after harvesting and storage for 3 months [30].

#### 4.2.4. Thermal Properties

From the thermogravimetric analysis (TGA), it is possible to report the temperature of thermal degradation and the proportion of each of the lignocellulosic fibre components (see Table 5). Initially, the evaporation of water and volatile components between 30 and 144 °C is presented, followed by the thermal decomposition of the hemicellulose at 178 °C, due to the presence of acetyl groups. When the fibre is exposed to a higher temperature, the cellulose is degraded to 296 °C, and finally, the lignin decomposes at high temperatures between 400 and 700 °C. As for its thermal conductivity, data of the order of 0.0253 W/m^2^ K are found, being a low value and suitable for the development of materials with an adequate thermal insulation capacity [30].

### 4.3. Methods of Extraction of Lignocellulosic Fibres from the Musaceae’s Pseudostem

**Figure 2:**
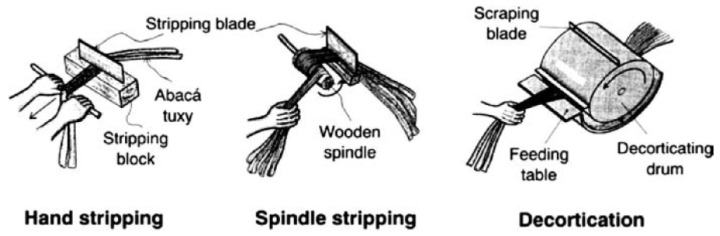
Extraction systems for lignocellulosic fibres from musaceae pseudostems. Figure reproduced with permission from reference [54]; copyright 2010 John Wiley & Sons Books.

When harvesting the bunch of a Musaceae, the pseudostem must be cut to prepare it for the lignocellulosic fibre extraction operation in the shortest possible time to avoid the development of decomposition from the high water content. The first stage focusses on the individual removal of each sheath, which is based on the generation of a cut between the external and intermediate sheaths, using a knife to facilitate the separation of each sheath from the pseudostem. In the second stage, a peeling system is required: manual, semi-manual, or mechanical, to separate the lignocellulosic fibres from the parenchyma, vascular tissue, stain or rubber, and water (see Figure 2). Peeling technologies are a mobile knife, manual fixed knife, and spindle peeling knife [28]. In most Musaceae, the fibres from the 11 sheaths closest to the outer part of the pseudostem (external and intermediate stage) are extracted because the fibres from the internal stage and the shell present low resistance to abrasion extraction [30].

#### 4.3.1. Mobile Blade

This technique can also be referred to as decortication or defibration, based on the use of a rotating drum fixed on a shaft. The blades are mounted on the drum body to generate the peeling shear during its rotation from the impulse of an engine. The shredder’s second element is a metal casing, which covers the drum and is located between 2 and 5 mm from the drum. When the drum rotates, each sheath is inserted into the shredder, generating a shear between the drum and one of the metal casing walls, allowing the lignocellulosic fibre to be extracted at one end of the sheath. The procedure is repeated on the opposite side of the sheath to achieve fibre-free impurities [30,54,63,66]. This technology’s production capacity is 80 to 100 kg/day, the quality of the fibre is low due to the limited degree of homogeneity, and significant economic investment is required [28].

#### 4.3.2. Manual Fixed Blade (Hand Stripping)

The sheath is placed between two blades that apply pressure to fix it. The position of the sheath between the two knives that apply pressure must be between 8 and 12 cm from one of the ends, being a sufficient space for the operator or farmer to manually pull the sheath to make the shear between the sheath and knives, achieving the extraction of the fibre. The procedure is repeated on the opposite side of the sheath to obtain the lignocellulosic fibres. The fibre extraction capacity is between 10 and 20 kg/day; the fibre’s quality is low due to the considered presence of impurities, and the economic investment is low [28].

#### 4.3.3. Spindle Peeling Blade (Spindle Stripping)

The fibres are pulled by a machine through a shaft or spindle rotation, generating the shear between two fixed blades and the sheath, allowing for adequate homogeneity, softness, and a low level of impurities in the extracted fibres, being the recommended technology for achieving greater quality. The knives must apply pressure to the sheath [28,54]. As the extracted lignocellulosic fibres are available in lengths of up to 3 m, they have a humidity of between 55 and 60%, requiring drying to a humidity of less than 10% for storage [28].

### 4.4. Pre-Treatment Methods for Lignocellulosic Fibres

The availability of lignocellulosic fibres makes it possible to develop various processed products such as fuel (e.g., alcohol, butanol), additives for the processing industry (e.g., glucose, xylitol), synthesis gas, or “Syngas” (e.g., methanol), polymer synthesis (e.g., polylactic acid), and the development of new fibre-reinforced or biocomposite materials, among others [50,67,68,69,70]. In the case of the development of composites and biocomposites, it is undesirable to use native lignocellulosic fibres in a polymeric matrix because the fibres have a hydrophilic character due to the presence of hydroxyl groups and C-O-C bonds in their structure. In contrast, several polymeric matrices have a greater hydrophobic character, generating limited interfacial adhesion and, consequently, they have low mechanical properties [71]. Following the above, the use of unitary methods and operations is required to modify the lignocellulosic fibres’ surface area, increasing interfacial compatibility with a defined matrix to obtain the development of a composite and/or biocomposite with increased resistance to tension, compression, bending, and rigidity. The degree of stress transfer from the matrix to the fibres can be increased [72]. The recommended pre-treatments of lignocellulosic fibres (physical, chemical, and biological) are mentioned below, and in some cases, combinations of methods can be made to increase efficiency in the separation of lignin, hemicellulose, amorphous cellulose, pectins, and waxes [67]. These types of surface modifications have been reported in research where the development of biocomposites from lignocellulosic fibres and thermoplastic and thermostable polymeric matrices derived from petroleum are proposed [50,67]. However, the use of natural matrices such as starches, tannin-based resins, and lignin, among others, is currently on the rise [73].

#### 4.4.1. Physical Methods

The methods that make up this group are related to changes in the fibres’ structural and surface properties, influencing the mechanical adhesion with the matrix used in the biocomposite; however, no changes in the fibres’ chemical composition are evident [50,72,74]. The most commonly used methods are grinding, corona treatment, plasma treatment, and steam explosion.

- Grinding. This is based on reducing the lignocellulosic fibres’ particle size when desired to produce a biocomposite by implementing a reinforcement with short fibres that achieve lengths of between 0.2 and 2 mm. The use of short fibres increases the specific surface area and reduces the degree of polymerisation and crystallinity of the cellulose. However, this adaptation method must complement a second method to contribute to the adhesion or mechanical anchorage with the matrix. Ball, vibrating balls, discs, or hammers mills can be used in the milling process [67,75,76].
- Corona treatment. From the corona discharge application on the fibre’s surface, a surface oxidative activation is generated, and its polarity increases, allowing a greater degree of compatibility between the hydrophilic fibres with hydrophobic polymeric matrices, especially when using polymers derived from petroleum. By achieving an adequate exposure time and intensity of the corona discharge in the lignocellulosic fibres, increases in the modulus of elasticity and maximum resistance to tension can be obtained. However, if the exposure time is increased to values greater than 15 min, the tenacity is reduced, and the degree of polymeric degradation of the lignocellulosic fibres increases [72].
- Plasma treatment. This procedure is similar to corona treatment based on exposing the lignocellulosic fibres to an electrical discharge, achieving a surface modification. However, for its adequate execution at low temperatures and exposure to atmospheric pressure, it is required to handle a greater number of process variables such as the type of gas to be used (e.g., oxygen, helium), type of frequency (radiofrequency or low frequency), flow, pressure, and concentration or plasma power [50]. Reactive free radicals are produced, as well as variations in surface energy, the generation of surface cross-links, and the development of the hydrophobic character of lignocellulosic fibres [50,72].
- Steam explosion. Lignocellulosic fibres are exposed to saturated water vapour at a temperature of 160 to 290 °C and a pressure of 0.70 to 4.85 MPa for 1 to 60 min in a closed system such as a reactor. Water is the most commonly used solvent; however, changing it to sodium hydroxide (NaOH), sulphuric acid (H_2_SO_4_), sulphur oxide (SO_2_), and sodium hypochlorite (NaClO) solutions increases the intensity of the operation [75,77]. When the fibres are exposed to the solvent at high pressure for short periods and subsequent decompression, the fibre structure explodes [77]. The macromolecule detached with the highest proportion of fibres is hemicellulose, which is hydrolysed and solubilised in water from simple sugars, mainly glucose and xylose. Its structure is altered for lignin, and it is removed in low proportions in lignocellulosic fibres [67,69]. If it is intended to increase the amount of lignin extracted, a temperature higher than the glass transition temperature of lignin (142 °C) should be considered during the unit operation’s execution to obtain a higher fibre surface roughness and increased crystallinity index [77,78].

#### 4.4.2. Chemical Methods

In this group of treatments, a chemical substance of a different nature to the lignocellulosic fibres is required to increase the degree of interfacial adhesion with the polymer matrix, since, using a physical blend of previous components of a biocomposite; the links generated are weak. The use of chemical treatment, apart from increasing interfacial interaction by generating primary (covalent) and/or secondary bonds, makes it possible to reduce the hydrophilic behaviour of the fibres [9,50,51] and to modify the chemical composition [50,72]. The most widely used methods are silanisation, alkalinisation, maleated coupling agent, and acetylation. However, other surface modification treatments of the fibres include benzoylation, peroxidation, sodium chlorite, acrylic, acrylonitrile coupling agent, isocyanates, stearic acid, oleoyl chloride, permanganate treatment, and triazine [50,72,74,75].

- Silanisation. Silanes are multifunctional molecules that are used as coupling agents to form covalent bonds called siloxane bridges, with phenyltrimethoxysilane being one of the most widely used, due to its high efficiency in generating bonds with lignocellulosic fibres (hydrophilic character) and the matrix (hydrophobic character); however, other coupling agents are found such as epoxy and urethane silanes [72]. Initially, the cellulose presented in the lignocellulosic fibre is modified through a chemical reaction by condensation between the silanol group belonging to the coupling agent and the hydroxyl group found in the cellulose, generating the Si–O–cellulose bond. In contrast, the other end of the coupling agent reacts with the matrix, generating the Si–matrix bond. Subsequently, as it has a modified fibre, its surface’s polarity is reduced, facilitating its mixture with polymeric matrices of a more hydrophobic nature. It also contributes to the reduction of porosities of the fibres from the coating of the coupling agent. This method generates biocomposites with an increase of mechanical resistance greater than that provided by alkalisation and acetylation [74].
- Alkalisation. This is the least complex and least costly treatment, using sodium hydroxide solutions between 2 and 15%, requiring immersion times of between 2 and 24 h and temperatures of between 60 and 120 °C to modify the surface of the lignocellulosic fibres [9,75] by breaking hydrogen bridges between the cellulose and other molecules, facilitating the release of significant portions of lignin, hemicellulose, waxes, pectins, and oils that cover the external cell walls, contributing to surfaces with greater roughness. It has also been shown that the hydroxyl groups (-O-H) in the cellulose are broken or altered, creating more reactive groups (-O-Na) and reducing the hydrophilic nature of the cellulose present in the modified fibre [74]. If the fibres’ immersion time in high sodium hydroxide concentrations is prolonged, damage or cracks may be generated in the fibre. In contrast, with an adequate concentration of alkali, the fibre diameter is reduced, favouring interfacial adhesion with the matrix, since the surface area and the aspect ratio (length/diameter) are increased [50].
- Coupling by maleation. Maleation coupling agents such as maleic anhydride generate C-C bonds between the surface of the lignocellulosic fibre and the polymer matrix. Two types of chemical reactions are generated in the maleic anhydrous: (a) between the maleic anhydrous and the hydroxyl groups of the lignocellulosic fibres; (b) between the maleic anhydrous and the polymeric matrix. One of the alternatives for carrying out the chemical reaction consists of melting the polymeric matrix with 0.5% maleic anhydrous and then coating or mixing the maleic matrix with the lignocellulosic fibres, allowing the generation of links with the hydroxyl groups coming from the cellulose and contributing to greater mechanical resistance and a reduction in the absorption of water in the biocomposite [79].
- Acetylation. The use of acetic acid and acetic anhydride is required to modify the lignocellulosic fibre’s surface, generating a hydrophobic character by incorporating acetyl groups (CH_3_CO) in the hydroxyl groups presented in the cellulose. Initially, the lignocellulosic material must be immersed in acetic acid. The acetic anhydride is added during an immersion time of between 1 and 3 h at a high temperature to accelerate the esterification’s chemical reaction between the hydroxyl group and the anhydrous group. The level of modification can be quantified by the degree of acetylation, with 18% being the maximum value permitted, since there have been considerable reductions in the degree of polymerisation and crystallinity of the cellulose contributing to the reduction of the tensile maximum resistance. However, acetylation values greater than 18% contribute to strengthening the modified fibre’s hydrophobic character [50,72,74]. This type of surface modification provides greater hydrophobic character and tensile strength in banana pseudostem fibres than that generated in plasma treatment [64].

#### 4.4.3. Biological Methods

From the action of white-rot fungi such as Phlebia radiata, Rigidosporus lignosus, and Jungua separabilima, enzymes are produced that degrade lignin, hemicellulose, and polyphenols. Brown-rot fungi are also found, presenting a specificity in the degradation of cellulose. Following the above, white-rot fungi can contribute to obtaining a modified fibre for the development of biocomposites due to the capacity to degrade lignin from peroxidases, polyphenol oxidases, manganese-dependent peroxidases, and laccases. However, the conversion speed is slow to implement on an industrial scale, as two weeks are required for adequate development of fungi and splitting of lignin. On the other hand, there is no optimum control in the specific splitting of lignin, as the enzymes can partially degrade the cellulose, and it is recommended that this method be used in lignocellulosic sources with a low lignin content [67,74].

#### 4.4.4. Influence of Pre-Treatment Methods on Lignocellulosic Fibres from Musaceae Pseudostem

After knowing the surface modification methods of greater use in lignocellulosic fibres, the methodologies used in banana and plantain fibres are reported with their respective mechanical, thermal, and physicochemical characterisations.

- Steam explosion. This physical technique on fibre from the banana pseudostem generates an increase in the cellulose’s thermal degradation temperature, going from 390 (native fibre) to 400 °C [65]. In a second study of the steam explosion in lignocellulosic fibres from banana pseudostem, an autoclave was used at a temperature of 220 °C, evaluating the structural changes of the fibres when exposed to high-pressure water vapour, using two operating times, 2 and 4 min. When comparing the holocellulose content (cellulose + hemicellulose) of the native fibre with its modified state, reducing its content was evidenced, going from 57.5 to 52.8%, due to the removal of the hemicellulose and amorphous cellulose in the fibres. Simultaneously, the proportion of lignin was increased, starting with a value of 20.3% in its native state until reaching a content of 23.2% when achieving the surface modification of the fibres when exposed for 4 min. By establishing a longer operation time, greater severity of the physical operation on the fibres is established, evidencing greater roughness through Scanning Electronic Microscopy (SEM), a greater index of crystallinity, and the degree of polymerisation of the cellulose is reduced [54,80]. However, replacing water with a 2% NaOH solution in an autoclave for 1 h at a temperature between 110 and 120 °C showed an increase in cellulose content from 64% in its native state to 82.4% in its modified state and a reduction in hemicellulose and lignin from 18.6 to 13.9% and 4.9 to 3.6%, respectively [77].
- Plasma treatment. A surface treatment equipment with plasma technology was used to carry out the surface modification on plantain pseudostem fibres, using the following conditions: ambient temperature, atmospheric pressure, ceramic electrodes with a potential discharge supply of 1 kW, speed of 4 m/min, and variation in dosage of 1, 3, and 6 kW min/m^2^. The modified fibres were characterised by using the tensile test, FT-IR spectroscopy, thermogravimetric analysis, and contact angle. In the FT-IR analysis, the formations of the 2850 and 2900 cm^−1^ bands are shown, relating a C-C transformation, contributing to a hydrophobic character in the fibres. The thermal stability of the cellulose shows an increase from 336.3 °C in the native fibre to 337.1 °C in the fibre exposed to a dosage of 1 kW min/m^2^, 342.1 °C at 3 kW min/m^2^, and 339.1 °C at 6 kW min/m^2^, identifying an increase in the mentioned property between 0.2 and 1.7%. The toughness is reduced in the tensile test when the modification is made, going from 0.3 (native fibre) to values between 0.20 and 0.27 N/Tex. At the same time, the contact angle is increased, going from 92.2° in the native fibre to values between 97.5 and 106.8°, being the superior value of the angle belonging to the dosage of 6 kW min/m^2^, contributing to the increase of the hydrophobic character in the fibre [64].
- The blend of alkalisation with peroxidation. The fibre of the pseudostem from the banana tree (10 g) was exposed to different solutions to remove the non-cellulose components, starting with an immersion of the fibre in a solution of sulphuric acid (H_2_SO_4_) at 55 °C for 2 h to remove the external wax, followed by a wash with distilled water to remove residual H_2_SO_4_. The second immersion consisted of using a solution composed of 200 mL of hydrogen peroxide (H_2_O_2_) with a concentration of 7 g/L, 3% of sodium silicate (Na_2_SiO_3_), and 2% of sodium polyphosphate at 95 °C for 1.5 h to remove hemicellulose and lignin. The third immersion consisted of the use of 200 mL of sodium hydroxide (NaOH) solution with a concentration of 9 g/L boiling for 3 h, and then, a wash was carried out using a solution of H_2_SO_4_ to neutralise the alkaline residues, and finally, the modified fibres were dried at 105 °C for 24 h. When evaluating the tensile properties of the modified fibres compared to their native state, the removal of lignin and hemicellulose contributed to the increase in the packing of the cellulose, generating an increase in the maximum tensile strength from 210 to 333 MPa, while the modulus of elasticity was reduced from 26.86 to 22.56 GPa, and the deformation at the breakpoint increased from 0.8 to 1.6%. Concerning thermal stability, the cellulose’s thermal degradation temperature increased by 10 °C due to its concentration in the modified fibres. From X-ray diffraction, the crystallographic pattern consisted of two peaks at 16 and 22.5° 2θ. Simultaneously, the crystallinity index of the native fibre presented a value of 56.6%, achieving an increase of 61.2% when performing the chemical modification due to the removal of amorphous structures represented in the hemicellulose [61].
- Acetylation. Two surface modification treatments were used on the plantain pseudostem fibres, the first being a blend of acetic anhydride and acetone at a ratio of 1:10 *w*/*w* and the second being a blend of acetic anhydride, epichlorhydrin, and acetone at a ratio of 1:1:20. The native fibres were submitted to immersion in the respective treatments for 24 h at 20 °C. Subsequently, the fibres were washed with acetone and distilled water to remove chemical residues. Then, the fibres were dried in an oven at 105 °C for 24 h. The modified fibres were characterised from the tensile test, FT-IR spectroscopy, thermogravimetric analysis, and contact angle. From the FT-IR analysis and comparison between the native fibres and the modified fibres, a reduction of the absorbance presented in the 3330 and 3600 cm^−1^ bands was identified in the modified fibres, generating a greater reduction in the fibres exposed to acetic anhydride and epichlorohydrin; it is possible that chemical reactions were generated in a more significant number of hydroxyl groups present in the fibre to establish esterification by the acetic anhydride and alkylation by the epichlorohydrin. In addition, bands of 3700 and 3850 cm^−1^ are evident in the modified fibres, relating the presence of -CH_3_ groups due to acetylation and bands of 2850 and 2900 cm^−1^ due to alkylation to give a greater hydrophobic character to the fibres. The thermal stability of the cellulose presented an increase from 336.3 °C in the native fibre to 359.3 °C in the fibre exposed to acetic anhydride, giving an increase in the mentioned property of 6.8% due to the increase of the cellulose content in the fibre, while the use of epichlorohydrin and the blend of acetic anhydride and epichlorohydrin reduce the degradation temperature to 329.5 and 328.6 °C, respectively. The toughness is reduced in the tensile test when the modification is carried out, going from 0.3 (native fibre) to values between 0.16 and 0.25 N/Tex. Simultaneously, the contact angle is increased, going from 92.2 ° in the native fibre to values between 116.3 and 133.14 °, the higher value given by the acetic anhydride and epichlorohydrin blend, due to the greater hydrophobic character obtained in the fibre [64].

## 5. Development of Biocomposites Made up of Lignocellulosic Fibres

The use of lignocellulosic fibres as a raw material for obtaining processed products generates different physical, thermal and mechanical properties in thermal and sound insulation panels, geotextiles, and composites [54]. In the case of composites, initially, mineral fibres such as glass (E-glass), carbon nanotubes, clays or graphite, and polymeric fibres derived from petroleum were included, with aramid, nylon, and rayon as reinforcements (discontinuous phase) in blends with matrixes based on synthetic polymers derived from petroleum (continuous phase) to provide greater mechanical and thermal resistance. However, as they fulfilled their functions in various engineering sectors, the composites were discarded in sanitary landfills or were incinerated, causing pollution in the environment. Under this, research has been carried out on new developments involving matrices and reinforcements from natural, renewable sources and with a biodegradable character to obtain biocomposites or green composites of lower density [55,81]. One of the alternatives is using lignocellulosic fibres, which are environmentally friendly, to replace glass fibres and other reinforcing fibres used in composite engineering [61]. Next, we will report on the different matrices and reinforcements used, type of orientation in the reinforcement, processing techniques, mechanical, thermal, and physicochemical properties of significant relevance and applications of the biocomposites, including those that have required some of the by-products coming from the musaceaes plants.

### 5.1. Biocomposites

**Figure 3:**
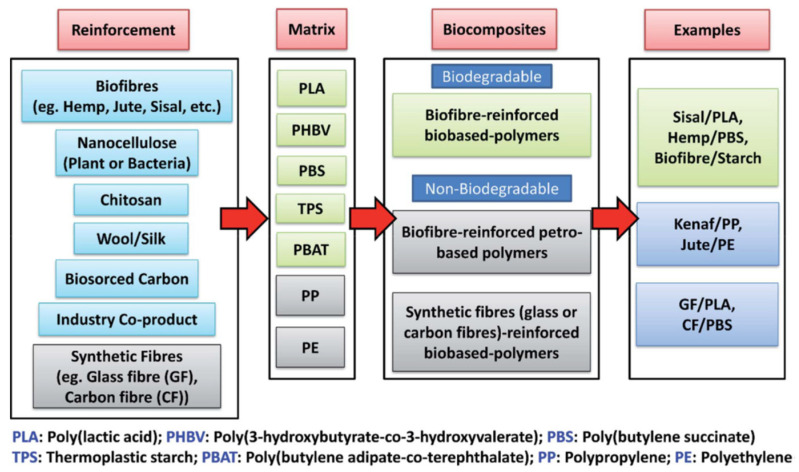
Types of biocomposites and examples. Figure reproduced with permission from reference [83]; copyright 2020 RSC Publishing.

Although some research defines the word biocomposite as those blends made up of lignocellulosic fibres and synthetic polymers derived from petroleum [50,54], the tendency is to use constituents from renewable sources with the capacity to be composted at the end of their functional life without affecting the environment (see Figure 3) [55,82,83]. Among their components are lignocellulosic fibres that provide reinforcement or filling functions, coming from wood sources (harvest and by-products) and non-wood sources (by-products of agriculture and agro-industry) [55]. Non-wood fibres provide a higher cellulose content, crystallinity, and lower density, generating greater interest in their introduction into different transformation processes at an industrial level [50]. The second component corresponds to the polymeric matrix from synthetic polymers derived from petroleum and/or biodegradable polymers, allowing an adequate distribution of the discontinuous phase and transmitting the stresses received by the composite’s matrix to the fibres to increase their mechanical resistance. To achieve this, it is essential to implement physical, chemical, or biological pre-treatments to increase the interfacial compatibility between the fibres and the matrix [55]. When comparing the biocomposite with the composite, several advantages are evident: lower production costs, less weight, greater flexibility, obtaining raw materials from renewable sources, biodegradability, thermal and sound insulation, non-toxic, reduced energy consumption during processing, no waste is generated when the material is incinerated, and it is not allergenic, while its disadvantages correspond to its lower values in its mechanical properties (especially on impact), high absorption of humidity, low durability, low resistance to fire and action of microorganisms, variations in its quality, limit in the processing, and application or operation temperature [50]. The long-term durability behaviour of biocomposites has not yet been considerably developed. It is a focus on additional research since the matrices and fibres used to manufacture them can degrade in aggressive environments such as exposure to ultraviolet (UV) electromagnetic rays, high humidity, and temperatures that promoting their aging and shorten the biocomposites useful lifetime in the different application sectors: aerospace, automotive, military, electronics, construction, packaging and containers, medical, sports equipment, and railways [50,83].

#### 5.1.1. Reinforcements

Different procedures have been used on lignocellulosic fibres to develop composites and biocomposites, considering the type of fibre orientation, type of surface modifications, fibre dimensions, and cellulose crystals to increase the reinforcing character, contributing to their mechanical, thermal, and physicochemical properties. In the fibre orientation in biocomposites, we find the use of nanocellulose, short fibres, unidirectional fibres, and fabrics.

Nanocellulose. Obtaining fibres with a high proportion of cellulose and dimensions in nanometres (nm) can be achieved from different raw materials that provide cellulose by using different methodologies. Wood and agricultural by-products can be used to obtain nanocellulose. However, the latter has a lower lignin content, and the cellulose microfibres are less packaged, demanding less energy during processing [84]. Several alternatives are available regarding the choice of methodology: bacterial synthesis, electro-nanofibre formation, mechanical treatment, biomechanical treatment, chemical–mechanical treatment, and chemical treatment [54]. Depending on the methodological choice, a characteristic diameter, length, and aspect ratio is obtained, classifying the nanocellulose group into microfibrils, microfibrillar cellulose, and cellulose whiskers. Microfibrils or nanofibres can be obtained from higher plants, identifying variations in their diameter according to their botanical source, reporting values between 2 and 10 nm, length greater than 10,000 nm, and an aspect ratio greater than 1000. Microfibrillary cellulose consists of aggregates of cellulose microfibrils with diameters between 10 and 40 nm, length over 1000 nm, and an aspect ratio between 100 and 150, while cellulose whiskers are obtained from the hydrolysis and sonication of microfibrils, generating chain cuts in the amorphous regions, giving diameters between 2 and 20 nm, a length between 100 and 600 nm, and an aspect ratio between 10 and 100 [84].

Short fibre. The fibre is obtained from the milling of longer filaments or a transformation process as a by-product, such as spinning. Since it has characteristic short fibre dimensions, it can be subjected to surface modification. When mixed with a matrix, a random orientation is obtained. The tensile strength depends on the degree of adhesion with the matrix, while the orientation contributes to the magnitude of the modulus of elasticity. Similarly, the critical length and aspect ratio must be considered [80].

Unidirectional fibre. Fibres with lengths equivalent to one of the composites or biocomposite dimensions are used; they are in a native state or with a modified surface and provide greater resistance to tensile and flexion in one of the dimensions of the composite [85].

Fabric or laminated fibre. The fibre reinforces two or more dimensions of the composite or biocomposite, with the option of using fibres with surface modification. This orientation can provide greater mechanical and thermal properties concerning the previously mentioned fibre orientations [85].

#### 5.1.2. Matrices

Its use in biocomposites consists of supporting the reinforcement material, being essential to establish an interfacial adhesion between the matrix and the reinforcement in order to facilitate the transfer of efforts homogeneously from the matrix to the fibres, and its resistance to the applied effort must avoid the propagation of cracks or damages in the biocomposite. In biocomposites, synthetic polymeric matrices derived from petroleum, agro-polymers, polymers of microbial origin, and biodegradable synthetic polymers can be used [50,82,83].

Synthetic polymers derived from petroleum. In this first group of polymers, they can be used in their thermoplastic or thermostable state. The polyolefins are represented by polyethylene, polypropylene, polystyrene, and polyvinyl chloride, and they are used as thermoplastic matrices in most cases, while epoxy, polyester, vinylester, and phenolic polymers are used as thermostable matrices [50]. In this case, it is essential to carry out a surface modification of the matrix and/or fibres to increase interfacial adhesion by using one or more physical and/or chemical methods [55].

Agro-polymers. This second group includes starch, pectin, and proteins. One of the most used polysaccharides is thermoplastic starch (TPS), which is constituted by native or modified starch, water, plasticiser, and minor components (additives) [86]. Plasticisers form hydrogen bridges with starch at high temperatures, generating strong interactions with the starch molecules’ hydroxyl group. When a plasticiser is added, the original hydrogen bonds (secondary bonds) between the hydroxyl groups of the starch molecule are destroyed, forming new hydrogen bonds with the starch, allowing the starch to be exposed to the plastification or mobility of polymer chains [6,87].

In the development of biocomposites, plasticisers are used that fulfil special functions of plasticising, such as lubricating, preventing hardening and crystallisation or retrogradation, among other vital functions for achieving a stable thermoplastic structure [88,89]. Among the most commonly used plasticisers are polyols: glycerol, mannitol, sorbitol, and xylitol [90,91]. Thermoplastic starch can absorb moisture, where the plasticiser and water contribute to this phenomenon, which generates changes in the material’s thermal and mechanical properties over time [54]. However, the water content and interactions in starch-based materials are dependent on the plasticiser concentration and the storage relative humidity [88,92]. New starch-based materials have been developed in recent years because of their biodegradable properties and because they are obtained from renewable sources [93]; however, these biomaterials are susceptible to the retrogradation recrystallisation of starch [94]. The term retrogradation describes a structural change that occurs in a starch after its gelatinisation, which initially presents an amorphous structure until obtaining a structure with greater ordered crystalline proportions as time goes by. These changes are due to the re-association of the starch’s polymer chains in a double helix structure [93,95]. Thermoplastic starch presents variations in its mechanical, thermal, physicochemical, and structural properties concerning the source of starch extraction used, including the type of species or variety. In the case of thermoplastic plantain starch with 40% glycerine content and processed by extrusion, it presents a tensile strength of 13.6 MPa, a modulus of elasticity of 0.4 MPa, and elongation at the breakpoint of 9.0% [96]. Meanwhile, other botanical sources such as rice, cassava, and corn have lower TPS tensile strength values than plantain TPS, while their corresponding modulus of elasticity is higher (see Table 6), respectively.

By adding lignocellulosic fibres in a TPS (matrix), a high degree of interfacial adhesion and compatibility is obtained in the biocomposite due to the structural affinity between the matrix and the reinforcement from the presence of hydroxyl groups in the two types of macromolecules [99]. On the other hand, changes in mechanical, physicochemical, and structural properties are generated in the TPS-based biocomposite, such as increased tensile and thermal strength, reduced water absorption, and restriction of starch retrogradation, due to the strong interaction of hydrogen bridges between starch and lignocellulosic fibre [55].

Proteins can be isolated from natural products such as cereals to obtain gluten (wheat) and zein (maize), while globulin, albumin, prolamin, and glutenin from legumes (peas and soybeans), as well as from animals such as casein, whey, keratin, collagen, and gelatin. In the case of soy protein, its structure is globular with the ability to solubilise in water; however, when denatured during a processing technique, it can develop rigid packaging, use as a coating, and the ability to be printed with inks. Limitations include low tensile strength and high water absorption, but by incorporating lignocellulosic fibres to fulfil the reinforcing function in a biocomposite, these limitations can be corrected [55].

Polymers of microbial origin. The polymers called polyhydroxyalkanoates (PHA) consist of polyesters synthesised and stored by bacteria that consume some substrates such as sucrose, starch, cellulose, and triglycerides [82]. The most commonly used copolymers are polyhydroxybutyrate-co-hydroxyvalerate (PHBV), polyhydroxybutyrate (PHB), polyhydroxybutyrate-co-hydroxyhexanoate (PHBH), and polyhydroxybutyrate-co-hydroxyoctanoate (PHBO). Some common limitations in this group of polyesters are reported, such as their brittleness and low thermal stability (200 to 250 °C) [55,91]. One of the alternatives to reduce brittleness is the application of citrates as a plasticiser in PHB, giving a maximum tensile strength of 22.7 MPa, modulus of elasticity of 3.7 GPa, and deformation at the breaking point of 6.62% [82]. Another way to mitigate the aforementioned limitations is by blending with lignocellulosic fibres to form a biocomposite, achieving higher crystallinity, thermal stability, and mechanical strength, due to interfacial compatibility between the matrix and the lignocellulosic reinforcement [55,91].

Biodegradable synthetic polymers. A group of thermoplastic polyesters from natural agricultural sources such as polylactic acid (PLA) and petroleum derivatives such as polycaprolactone (PCL) and polybutylene adipate-co-terephthalate (PBAT) are found; however, an aliphatic polyester such as polybutylene succinate (PBS) is obtained in either of the two previously mentioned sectors [6,83,100]. One of the most widely used polyesters is polylactic acid, which is acquired from the processing of starches and sugars from various primary agricultural products such as rice, sugar cane, sugar beet, potato, and corn. Carbohydrates must undergo hydrolysis (optional), lactic fermentation, lactic acid purification, and polymer synthesis from direct condensation polymerisation or ring-opening polymerisation of the lactide (cyclic dimer) [52,101]. PLA, being an aliphatic polyester, can replace several petroleum-derived polymers due to its high modulus of elasticity (1.2 GPa), high tensile strength (39.3 MPa), low water absorption capacity (0.4 to 0.6%), and gas barrier (O_2_, CO_2_, and H_2_O). However, it has the disadvantage of being brittle, low capacity to absorb energy on impact, and low degradation temperature [50,52]. By mixing PLA with lignocellulosic fibres to obtain a biocomposite, the modulus of elasticity and tensile strength can be increased; however, there is a limit to the addition of fibre with values close to 30%, since, when exceeding it, the tensile value is reduced due to the weak interaction between the matrix and the reinforcement [55]. 

#### 5.1.3. Processing Techniques

Despite the evidence in the development of research related to the production of biocomposites since 2001, the main focusses have been on the chemical modifications in the surface of the reinforcing fibres, reduction of water absorption, and increase in the properties of tension and flexion; however, there are limited scientific developments related to increasing the diversity in processing techniques for obtaining biocomposites, increasing resistance to flame and impact [50]. 

The processing parameters that should be considered for the production of the biocomposite are the type of fibre (length, aspect ratio, and chemical composition), the proportion of fibres to be incorporated to increase stiffness, mechanical resistance, and reduction of deformability, and the processing temperature should be less than 200 °C to avoid thermal degradation of the lignocellulosic fibres used as reinforcement. The most commonly used processing techniques are compression moulding, extrusion, injection moulding, and resin transfer moulding techniques [50,82].

Compression moulding. This is one of the most straightforward processing techniques; it has been used since the beginning of the 1990s to process composite materials, based on the use of a set of moulds with a cavity that allows the storage of polymeric matrix blended with fibres and/or fillers. The biocomposite’s mechanical behaviour depends on the proportion (up to 40%) and the reinforcement location. The reinforcement’s characteristics in the processing technique can have short fibres with random orientation, long fibres oriented in a unidirectional way, or fabrics (woven); the last two orientations generate greater values in the mechanical properties [102,103]. It is important to have a preheating stage of the molds to establish a molding operation in less time. The moulds compress and deform the biocomposite using high pressure for a determined time; later, the moulds are cooled, maintaining the pressure until solidifying the biocomposite. Among the essential processing parameters in operation are the amount of material to be cast, heating time, the pressure applied to the cast material, and cooling time. The disadvantages found in this technique are low production speed and the processing of biocomposites with flat surfaces and shapes until slight curvatures are achieved [50].

Extrusion. Extrusion is a method used for the generation of a high orientation in biocomposites [104]. The extrusion operation aims at mixing, transporting, melting, and forming processed biocomposites [105]. This processing technique is available to produce fibres, films, tubes, and biocomposites, among others. Extrusion is based on the deformation of pellets or powders based on thermoplastic polymers using temperatures above the melting temperature or amorphous polymers above the glass transition temperature. It is recommended to incorporate short fibres in the extruder’s second feeding point, achieving its mixing with the melted matrix [52,106]. The deformation generated in the extrusion is through conical dies, giving shape and modifications in the structure of the polymeric materials of different natures [107]. The disadvantages identified in this technique are its uniform cross-sectional area and low precision in the dimensions obtained in the biocomposite [50]. For the production of the polymers, the extrusion ratio, the pressure, the screw speed (r.p.m.), the temperature of the three or more zones, and the die geometry must be taken into account, as these are influential parameters in the production process, structure, and properties of the polymers [104,105]. In polymers’ extrusion, polymers are obtained with a structure that provides high anisotropy properties, which are manifested through mechanical resistance and deformation, thermal expansion, thermal conductivity, and gas permeability. This anisotropy is connected with the chains’ orientation and the reconstruction of the crystalline and amorphous regions [104]. Apart from obtaining solid bodies in TPS blends with lignocellulosic fibres with evident interfacial compatibility, it is also possible to get biocomposites in foam, using TPS as a matrix and with a maximum fibre content of 10% [102].

Injection moulding. Biocomposite pellets obtained previously by single or twin-screw extrusion are used, reporting the presence of short fibres distributed in the thermoplastic matrix. The pellets are introduced into the equipment, subjected to high temperatures above the melting temperature of the matrix and shear stresses generated by a conical die, achieving the ability to mould the biocomposite through its displacement or injection into a split die mould, cooling for a few seconds to harden, and releasing the biocomposite-based part. The type of reinforcement that can be used in this technique is short fibre [106]. In the case of biocomposites made from TPS, the fibre content used in this processing technique should not exceed 7% to achieve an increase in the biocomposite’s mechanical properties, and the fibre length should be between 60 µm and 1 mm [102]. The major disadvantage in this technique is the high economic investment in the operation setup, such as the price of the required moulds [50].

Resin transfer moulding techniques. These processing techniques are mainly used for biocomposites using thermosetting polymers as a matrix. Alternatives include hand lay-up, resin transfer molding, and resin infusion molding [50].

### 5.2. Biocomposites from Starches, Flours, and Fibres from Musaceae

Different types of biocomposites have been developed based on the Musaceae’s by-product, such as starches, flours, and/or lignocellulosic fibres from the pseudostem, using different types of matrix, surface modifications of the lignocellulosic fibre, fibre orientation, type of processing technique used, and resulting mechanical properties. Next, the different biocomposites will be reported according to the type of matrix used: synthetic polymers derived from petroleum, agro-polymers, polymers of microbial origin, and biodegradable synthetic polymers.

#### 5.2.1. Synthetic Oil-Based Polymers

A comparison was made between biocomposites based on lignocellulosic fibres from musaceaes pseudostem distributed in different polymeric matrices, low-density polyethylene (LDPE), polyester, and epoxy resin. In the first matrix, LDPE, three biocomposites were produced by compression moulding, which was made up of 40% long fibres (length 150 mm) with a unidirectional orientation. The differentiation of each biocomposite is due to the long fibre conditions, using native fibres (LDPE/BF), fibres modified from a methacrylate, benzoyl peroxide and methanol immersion at 70 °C (LDPE/MBF1), and fibres modified with methacrylate, benzoyl peroxide, methanol, and starch (LDPE/MBF2). In Table 7, an increase in the mechanical properties (tension and impact tests) is shown due to the increase in interfacial adhesion between the matrix and the reinforcement, especially when a 4% starch solution is incorporated in the modification method, generating an increase in the rigidity of the fibres. As for the water absorption of the biocomposites during 30 days, the LDPE/MBF2 biocomposite presented an equilibrium absorbing 5.6% of water, while the LDPE/MBF1 biocomposite did not achieve an equilibrium in the evaluated time, reaching 12.6%, due to the generation of greater spacing between the fibre and the matrix, since it is a biocomposite with a lower degree of interfacial interaction and greater availability of O-H groups in the native fibre, while the fibre modified with methacrylate, peroxide, methanol, and starch generated a coupling or covalent bond between the O-H groups of cellulose and polyethylene. In the case of using native fibres within the biocomposite, being the alternative with less interfacial adhesion, it manages to generate a reinforcement due to the lower tensile properties of LDPE, reporting a maximum tensile strength, modulus of elasticity, and elongation at break of 10.9 MPa, 178 MPa, and 386%, respectively [103].

Polyester resin as a second matrix was used to produce a biocomposite by compression moulding, using 15% of the plantain pseudostem’s short fibre as a reinforcing agent, which was subjected to a surface modification from acetylation (acetic anhydride and epichlorohydrin). Taking into account one of the tensile properties of polyester (maximum tensile strength of 15.1 MPa) and what is reported in Table 7, the reinforcement is evident by providing 15% of native short fibres; however, the acetylation in the fibres increases the interfacial adhesion between the fibres and matrix. Meanwhile, the water absorption of the polyester and the biocomposite (native fibres) after being immersed for 8 days reported values of 0.8 and 3.95%, respectively, showing an increase in water absorption in the biocomposite due to the presence of native fibres with a hydrophilic character, while the biocomposite with acetylated fibres reduced water absorption to 2.43%, due to the more significant hydrophobic character of the fibres by substituting the acetyl group in some O-H groups of the cellulose presented in the fibre [108]. Another investigation using the same polyester resin incorporated 4% of short fibres (length from 2 to 5 mm) belonging to the plantain pseudostem in its native state, which were alkalised to 10% and acetylated, reporting a reduction in the resistance to flexural strength in the biocomposites (63.1, 69.7, and 81 MPa) when compared with the polyester resin (94 MPa), due to the weak mechanical response in the interfacial adhesion between the fibres and the matrix [110].

In a third matrix, epoxy resin was mixed with short native and alkalised (1%) fibres and randomly oriented to obtain two types of biocomposites, evidencing an increase in the tensile strength, modulus of elasticity, and impact resistance (see Table 7). In the case of resistance and modulus of bending, the same trend was observed, increasing from 57.5 MPa and 11.81 GPa, respectively, in the biocomposite with native fibres to 69.0 MPa and 13.2 GPa, respectively, in the biocomposite with modified fibres [109].

According to the previous research, the alkalinisation treatment carried out on the musaceas fibres gave a more significant increase in tensile strength, modulus of elasticity, and impact resistance in biocomposites based on an epoxy resin matrix due to the removal of lignin, hemicellulose, and amorphous cellulose, which contributed to the physical anchorage from the surface roughness pattern generated in the fibres.

#### 5.2.2. Agro-Polymers

The incorporation of natural fibres in TPS-based matrices is intended to develop biocomposites with higher stability properties, as in tensile tests, generating increases in maximum tensile strength up to four times established in a TPS [102]. According to the literature review, no biocomposites are reported to be made from starch and/or flour from bananas or plantains, reinforced with lignocellulosic fibres from bananas or plantains. However, research has been conducted on biocomposites using fibres from the Musaceae’s pseudostem blend with TPS from corn and cassava.

TPS’s biocomposites from corn with 30% glycerin and randomly incorporating 50% of short fibres (length between 7.8 and 22.2 mm) from the plantain pseudostem alkalised with 0.5% NaOH through the use of the compression molding technique. The alkalised fibres have a tensile strength of 148 MPa, modulus of elasticity of 9.7 GPa, diameter between 57 and 249 µm, critical length (Lc) of 5.96 mm, aspect ratio (l/d) of 98, and density of 1350 Kg/m^3^. The processing conditions were an applied pressure of 5 MPa, temperature of 160 °C for 30 min, and subsequent cooling of the mould at a 2 °C/min rate. When comparing the tensile properties of the corn TPS matrix (maximum tensile strength of 3.8 MPa and modulus of elasticity of 378 MPa), the incorporation of fibres from the plantain pseudostem increased the respective properties considerably (see Table 8). In the fracture area, adequate adhesion between the fibres and the thermoplastic matrix was observed, explaining efficiency in the transfer of load from the matrix to the fibres due to the generation of a simultaneous fracture in the matrix, reinforcement, and interfacial area [111].

In a second research study, the obtaining of a biocomposite is related, keeping the corn thermoplastic starch with 30% glycerin (matrix) and 25% short native fibre (length from 3 to 5 mm) of plantain pseudostem randomly oriented (reinforcement), using the compression moulding technique, obtaining superior tensile properties concerning the TPS matrix [112]. However, this increase is less than that reported in the previous research due to alkalisation in the plantain pseudostem fibres (see Table 8).

In a third research study, a biocomposite was developed from cassava TPS with 40% glycerine mixed with 20% short native fibres of banana pseudostem with a length of 2 to 10 mm, using an extruder, operating at 40 rpm, three heating zones with an average temperature of 130 °C and a sheet die. The tensile properties related to maximum tensile strength and modulus of elasticity increased concerning that given by the cassava TPS matrix (4.7 and 110 MPa, respectively), while elongation at break decreased from 68 to 4.8% (see Table 8). Taking into account the thermogravimetric analysis (TGA), the fibres presented in the biocomposite increase the thermal degradation temperature from 275 to 300 °C, providing greater thermal stability [113,114], having the same process conditions and characteristics of the previous blend with the addition of 8.6% magnesium hydroxide (Mg(OH)_2_), cross-links formed in the starch, contributing to the considerable increase in maximum tensile strength and modulus of elasticity. At the same time, deformation at the breakpoint was reduced (see Table 8). On the other hand, Mg(OH)_2_ managed to transmit a flame-retardant effect in the biocomposite [113]. 

Considering the revisions of biocomposites consisting of starch and modified short lignocellulosic fibres from banana pseudostem, it is possible to obtain tensile properties close to those of biocomposites made from petroleum-derived resins. However, further studies are required to focus on the reduction of water absorption in order to mitigate losses in mechanical and thermal properties, since TPS can obtain a value close to 37% of the water absorbed, while blends with lignocellulosic fibres can reduce their absorption by 21 to 24% [115,116].

Using a thermoplastic starch matrix in the biocomposites, the alkalinisation of the Musaceae fibres, used as reinforcement, generated increases in tensile strength between 468 and 668%, while the incorporation of Mg(OH)_2_ generated an increase of 428%. In Young’s modulus, the same behaviour was also evidenced, increasing between 802 and 961% by alkalinising the fibres. However, the use of Mg(OH)_2_ achieved the highest increase with a value of 2718% from the crosslinking generated with the thermoplastic starch and the fibres.

#### 5.2.3. Polymers of Microbial Origin

The blend of some of the biopolyesters that make up the PHA family with natural fibres generates greater interfacial adhesion to that generated with biocomposites made up of synthetic polymers derived from petroleum such as polypropylene, since PHA can wet the fibres as evidenced in Scanning Electronic Microscopy (SEM). However, as the plasticiser content in the biocomposite increases, interfacial interaction is reduced. The biocomposite has a higher modulus of elasticity and lower maximum stress resistance in terms of tensile properties concerning the matrix, as in PHBV [102]. 

PHB is the biopolymer with the most important use in the development of new plastic products. The blend of the previous matrix with short (5 mm long) alkalised fibres (5% solution) from the banana pseudostem variety Prata was sequentially used in two processing techniques, subjecting the blend initially to extrusion with an average processing temperature of 160 °C at a screw speed of 120 r.p.m., after which the biocomposite bead was cooled in water immersion with a temperature of 30 °C and pelletised. Subsequently, the biocomposite pellets were subjected to compression moulding at a pressure of 3 MPa and a processing temperature of 160 °C for 4 min, obtaining two types of composites, differentiating with the fibre concentration: 5 and 10%. A reinforcing effect on the mechanical properties was evident in the tensile, bending, and impact tests of the biocomposite containing 5% fibre. However, when the fibre content was increased to 10%, the reinforcing effect was maintained to the PHB matrix but in a smaller proportion when compared to the biocomposite with 5% fibre, due to a lower interfacial interaction between the matrix and the fibres from the lack of fibre distribution in the matrix and generation of force centres (see Table 9) [117].

Similarly, the thermal degradation temperature was reported through thermogravimetric analysis (TGA) and the Shore D hardness, showing that the addition of fibre contributes to greater thermal stability, going from 270 °C in the pure PHB to values between 300 and 305 °C in the two biocomposites, while the hardness decreases as the fibre content in the biocomposite increases, going from 81.7 Shore D in the pure PHB to 72.3 Shore D in the biocomposite with 10% fibre [117].

#### 5.2.4. Biodegradable Synthetic Polymers

Several developments have been made, which have mainly focussed on using PLA as a polymeric matrix. It has outstanding mechanical, thermal, and physicochemical properties among the biodegradable materials, allowing it to replace synthetic polymers derived from petroleum in applications of greater demand and stability [118]. 

In a first development, a blend of PLA with 40% short modified fibres (length between 20 µm and 1 cm) from the banana pseudostem was used and subjected to a sequential transformation, initially using an internal rotation mixer at 170 °C with a stirring speed of 60 r.p.m. for 15 min. The biocomposite’s pellets obtained were subjected to compression moulding at a temperature of 185 °C in order to obtain sheets with a thickness of 0.5 mm. Finally, the biocomposite was cured at 130 °C for 1.5 h to generate a matrix’s crystallisation. The fibres’ modification consisted of the first stage of alkalinisation from immersion in a 4% NaOH solution for 45 min and subsequent silanisation from the triethoxy-vinylsilane. Table 10 shows an increase in the maximum resistance to tension, modulus of elasticity, and resistance to flexion of the PLA/FBM1 biocomposite concerning that presented by pure PLA (39.3 MPa, 1.2 GPa, and 39.4 MPa, respectively), due to the existence of interfacial interaction between the fibres and the matrix, promoting an increase in the rigidity of the biocomposite. In contrast, the addition of fibre in PLA reduced the elongation at the point of break and resistance to impact, from 2.5% and 22.2 J/m in pure PLA to 0.24% and 17.1 J/m in the biocomposite with 40% modified fibre, due to the reduction in the capacity to absorb energy on impact and greater rigidity in the biocomposite. The thermal degradation of PLA decreases as the fibre content increases from 383.7 °C in pure PLA to 362.6 °C in the biocomposite, as the fibres begin their thermal degradation at 200 °C [119].

In a second study, acetylation was used as a surface modification method in the fibre of plantain pseudostem by immersion in an acetic anhydride/acetone solution at a ratio of 1/10 for 24 h at room temperature. From the native and acetylated fibres, fabrics corresponding to the reinforcement were made, while two PLA matrices were used with the differentiation in their structure: amorphous and semi-crystalline. Following the above and its different interactions between the type of reinforcement and type of matrix, four types of biocomposites were obtained, being processed through compression moulding by using a processing temperature of 175 °C and applying pressure between the moulds from two stages: 3 Kg/cm^2^ for 6 min and 232 Kg/cm^2^ for 1 min when semi-crystalline PLA was used, while amorphous PLA required 175 °C, 4 Kg/cm^2^ for 4 min, and 232 Kg/cm^2^ for 1 min, respectively. The tensile properties were increased by incorporating the native fibre fabric concerning the pure PLA matrix, presenting a maximum tensile strength of 35 MPa and modulus of elasticity of 1350 MPa, while contribution to the reinforcing effect of acetylated fibres was less (see Table 10). The thermal stability maintains the same behaviour as the previous investigation, reporting a lower temperature of thermal degradation when adding fibres in the PLA, going from 350 to 319 °C [120].

In the previous research, an increase in the mechanical properties was related to incorporating native fibre from the Musaceae’s pseudostem. However, the opposite behaviour has been reported, evidencing the absence of interfacial adhesion between short fibres (length between 2 and 3 cm) with random orientation and PLA in a biocomposite made from a simple screw extruder to obtain the pellet of the biocomposite with 30% fibre and later, to obtain sheets employing injection moulding (see Table 10). The processing conditions in the extruder consisted of using an average processing temperature of 181.7 °C with three heating zones at a screw speed of 40 rpm, while those for injection moulding corresponded to a melting temperature of 170 °C, mould temperature of 40 °C, injection time of 5 s, and an injection pressure of 145 psi. The use of alkaline fibres in the biocomposite generated a 9.6% increase in the maximum tensile strength, 0.1% in the modulus of elasticity, and 3.1% in the impact resistance, evidencing a slight recovery of interfacial adhesion between the two phases. However, by adding 3% nanosilicates (C30B) in the biocomposite, the maximum tensile strength was increased by 242. 2%, the modulus of elasticity was increased by 2.2%, and the impact resistance was increased by 78.5% when using native fibres, while in the alkaline fibres, increases in properties were evident in 218.6, 9.7, and 74.7%, respectively. The blend between PLA and natural fibres in a melting operation contributes to generating interstitial spaces between the polymeric macromolecules involved. The participation of nanosilicates in the biocomposite is achieved by placing them in these spaces, promoting strong interactions between the matrix and the fibres through physical links with the carbonyl and C-O groups of PLA and links with the fibres through the formation of hydrogen bridges. Another advantage of nanosilicates is increasing the degradation temperature from 349.4 °C in pure PLA to 372 °C in the biocomposite with the nanoparticles. Another alternative contributing to the increase in thermal stability is coupling agents such as silanes [118].

From the evidence in Table 10, alkalinisation followed by silanisation of the fibres contributed to the higher tensile strength and Young’s modulus in PLA matrix-based biocomposites, generating increases of 100 and 500%, respectively. Meanwhile, incorporating alkalinised fibres and nanosilicates contributed to the highest impact strength (between 33.7 and 76.1%). On the other hand, regardless of the semi-crystalline or amorphous nature of the PLA used in the composite, no considerable increase in the tensile properties of the generated biocomposite was evidenced.

From the use of dynamical mechanical analysis (DMA), an increase in the storage modulus (E’) was identified in biocomposites consisting of PLA and banana fibres (30%), concerning the pure matrix, reaching values of 4.4 and 5.3 GPa with the use of native and alkalinised banana fibres, respectively. This is due to the increase in the interfacial interaction between the fibres and the polymeric matrix [121]. The behaviour was similar to that reported for a biocomposite consisting of PLA and 5% abutilon fibres, where E’ went from 1.5 GPa (pure PLA) to 1.8 GPa with the reinforcement [122]. 

Finally, we consider the previous reports of the different biocomposites based on lignocellulosic fibres from Musaceae pseudostems, which were blended with polymeric matrices of a different nature: synthetic polymers derived from petroleum, agro-polymers such as starches (from cereals, tubers, and roots), polymers of microbial origin, and synthetic biodegradable polymers; the high adhesion achieved between natural fibres and matrices based on TPS and PHB is highlighted. Regarding the tensile properties, the highest values of maximum strength and modulus of elasticity were defined when using fibres with surface modification in each of the matrices, highlighting the results reported for PLA (14.5 to 78.6 MPa and 284 to 7200 MPa, respectively). Their values are higher than those referenced for the other matrices. In the percentage by which the degree of reinforcement was increased, TPS presented the most significant increases in tensile strength when mixed with Musaceae fibres, generating increases from 210.6 to 668%, followed by LDPE (70.6 to 167.9%), polyester (83.4 to 103.3%), PLA (7.6 to 100%), and finally, PHB (8.8 to 13.9%). The matrices that can be used in biocomposites with the highest weathering resistance are synthetic polymers derived from petroleum and PLA, while in TPS, the implementation of coatings to reduce water absorption should be considered. The matrix with the highest thermal stability in the biocomposite is PLA.

### 5.3. Applications of Biocomposites

Currently, biocomposites based on lignocellulosic fibres have been implemented in the market according to the matrix’s properties and the type of reinforcement. The properties are classified as follows: (a) High mechanical resistance with low weight, implemented in automobile and aircraft parts, electronic equipment, sports elements, and storage tanks; (b) Renewal of products such as plastic covers, containers, toys, and household appliance housings; (c) Non-toxic, relating the manufacture of toys and products handled by the consumer; (d) Biocompatible, applied in medical devices and implants; (e) Low cost for the construction of furniture, sound, and thermal insulation panels, gardening products, and packaging; (f) High moisture absorption for the preservation of dry products; (g) Low durability, characteristic in products of single-use in the short term and sensitive to aggressive environments. However, the potential to be developed for advanced applications such as pipes, casings for electronic devices, structures, or elements that must be exposed to the weather and/or humidity is evident [50,55]. According to the literature review, limited applications have been reported in the automotive industry [123,124], orthopedic implants [125], containers, and packing [126,127,128,129]. As for the use of products from Musaceae plants, only one development was identified related to the elaboration of lids for rigid containers by implementing short lignocellulosic fibres from plantain pseudostem.

### 5.4. Containers and Packaging

Oil-based plastics have been used as materials for agricultural applications, rubbish bags, and market or food packaging, contributing to an annual production of 30 million tons of plastics in the packaging sector, which is a value equivalent to 25% of global plastics production, maintaining a growing consumption over time [123]. In one of the developments identified, a biocomposite was developed for the construction of a lid for rigid containers made of glass or metal for the storage of coffee, using a blend of 30% polylactic acid (PLA), 30% high-density polyethylene (HDPE), and 40% short fibres of the plantain pseudostem modified by acetylation, which was initially extruded to obtain the pellets of the biocomposite, and subsequently, the lid was obtained by injection moulding [130].

## 6. Future Perspectives

Considering that plantain gives higher yields in starch extraction, future research can focus on using this variety of Musaceae by-products to obtain thermoplastic starch. Likewise, greater use of the biomass from the pseudostem, leaves, and banana peels should be considered for the extraction of lignocellulosic fibres that reinforce the TPS in order to obtain new compounds completely bio-based on the by-products of Musaceae, with potential application as food utensils; addressing a sustainable model of circular economy, where these wastes are given greater added value by converting them into resources that can be introduced into production systems.

The good mechanical properties of the biocomposites reinforced with the previously modified Musaceae fibres constitute an opportunity to improve the performance of the bio-based composite. However, it is necessary to further develop and implement the most desirable surface treatment of the fibres in order to generate a better balance between the technical and economic conditions of their application while maintaining a low negative impact on the environment.

Since the extraction, modification, and processing processes necessary to obtain biocomposites from Musaceae by-products could affect their biodegradability and/or compostability, it is necessary to carry out biological studies on the materials in each of their different phases until they become a finished product in order to have a better overview of their conditions of use and the forms of disposal at the end of their useful life.

## 7. Conclusions

The starch, flour, and lignocellulosic fibres of the pseudostem from the different hybrid species of banana and plantain crops have the potential to be used in the elaboration of biocomposites due to their bioavailability, ease of incorporation in transformation operations when exposed to processing temperatures close to 200 °C, and shear stress. 

According to the root characteristics of the Musaceae, the banana has a greater capacity for absorbing nutrients in the soil than the plantain, allowing for the development of larger bunches, leaves, and stems. However, plantain gives a higher yield in starch extraction as long as the optimal agroclimatic conditions for its cultivation are met, depending on the species or variety used. On the other hand, the processing of by-products such as hulls and pseudostems for starch extraction and leaves for obtaining lignocellulosic fibres should be considered, allowing greater use to be made of the biomass belonging to the Musaceae crop.

Biocomposites consisting of musaceous fibres blended with bio-based matrices of different nature were reported, highlighting the generation of better adhesions in biocomposites based on TPS and PHB. Likewise, the highest tensile strength and modulus of elasticity were achieved in PLA matrices reinforced with surface-modified fibres. The most commonly used processing techniques for the transformation of Musaceae-based biocomposites are compression moulding, extrusion, and injection moulding. 

In the review, only one application was found in which plantain pseudostem fibres are used as reinforcement for the manufacture of biocomposites with matrices based on blends of PLA with HDPE, the material being used for the manufacture of lids for rigid containers for coffee storage. This suggests an essential outlook in developing formulations for new bio-based composites from Musaceae that can be used in other applications such as trays, plates, cups, and blending sticks.

## Figures and Tables

**Table 1:** Classification of bananas and plantains.

Genomic Group	Score	Species
AA Diploide	15–25	Bocadillo ^1^
AAA Triploide	15–25	Gros Michel ^1^, Cavendish Enano ^1^, Cavendish Valery ^1^, Dwarf Cavendish ^1^ Indio ^1^, Guineo ^1^, Cachaco ^1^, Morado ^1^
AAB Triploide	26–46	Hartón ^2^, Dominico Hartón ^2^, Maqueño ^2^, Pompo comino ^2^, Prata Ana ^1^
ABB Triploide	59–63	Topoco o Bluggoe ^3^, Pisang Awak ^1,3^, Pelipita ^2^, Popocho ^2^
ABBB Tetraploide	67–69	Klue Teparod ^3^
AAAB Tetraploide	67–69	Atan ^3^, Goldfinger o FHIA 01 ^1^, Prata Graúda ^1^
AABB Tetraploide	67–69	Kalamagol, FHIA 26 ^1^
BB Diploide	70–75	Abuhon ^2^, Pisang Wulung ^2^
BBB Triploide	70–75	Saba ^3^

Type of Musaceae; ^1^ Sweet banana; ^2^ Banana; ^3^ Cooking banana [7,14,19,21,22,23,24,25].

**Table 2:** Chemical composition of different sources of lignocellulosic fibres.

Specie	Cellulose (%)	Hemicellulose (%)	Lignin (%)	Moisture (%)	Others (%)	Author
Banana	31.3	14.9	15.1	9.7	4.46 (extractives) 8.65 (Ash)	[56]
Banana	62.5	12.5	7.5	N.R.	4.0 (Pectin)	[55]
Banana	64.0	19.0	5.0	10–11	N.R.	[9]
Plantain	56.8	11.8	19.1	10–11	1.3 (Extractives)	[9]
Nendran Plantain	59.3	10.2	17.5	9.1	1.0 (Ash)	[59]
Abaca	56–63	20–25	7–9	15	3 (Wax)	[50]
Cotton	85–90	5.7	0.7–1.6	1.0	0.6 (Wax) 0–1 (Pectin)	[52]
Coconut	32–43	0.15 a 0.25	40–45	3–4	N.R.	[52]
Abutilon	67–71	N.R.	17	N.R.	3.2 (Ash)	[58]

N.R.: Not reported.

**Table 3:** Physical properties of different sources of lignocellulosic fibres.

Specie	Diameter (µm)	Length (cm)	Author
Banana	56–143	N.R.	[61]
Banana Ambul	355	100–200	[62]
Plantain	80–250	N.R.	[30]
Nendran Plantain	50–250	N.R.	[59]
Abaca	114–400	250–350	[55]
Cotton	11–22	10.3–65	[50]
Coconut	100–460	35–62	[50,55]
Abutilon	11.4	8.5	[58]

N.R.: Not reported.

**Table 4:** Tensile properties of different lignocellulosic fibres.

Specie	Tensile Strength (MPa)	Modulus of Elasticy (GPa)	Tensile Strain (%)	Tenacity (N/Tex)	Author
Banana	210	26.86	0.8	N.R.	[61]
Banana	54–754	7.7–20	10.35	N.R.	[30]
Banana	800	32	3.7	N.R.	[60]
Nendran Plantain	182.33–631.74	N.R.	1.24–2.1	N.R.	[59]
Dominico Hartón Plantain	200–300	N.R.	1.9	0.47	[63]
Plantain	N.R.	N.R.	N.R.	0.30	[64]
Cotton	287–800	5.5–12.6	3–10	N.R.	[52]
Coconut	108–252	4–6	15–40	N.R.	[51,52]
Abutilon	N.R.	N.R.	2.5	N.R.	[58]

N.R.: Not reported.

**Table 5:** Thermal degradation temperature (TD) in lignocellulosic fibres.

Especie	TD Hemicellulose (°C)	TD Cellulose (°C)	TD Lignin (°C)	Author
Banana	N.R.	250–370 (65–71%)	200–500 (20–30%)	[61]
Banana	N.R.	260–390	400–500	[65]
Banana	178	296	501	[30]
Plantain	N.R.	336.3	>400	[64]

N.R.: Not reported.

**Table 6:** Tensile properties of thermoplastic starches.

TPS	Tensile Strength (MPa)	Modulus Of Elasticity (MPa)	Tensile Strain At Break (%)	Author
Rice (30% glycerol)	1.8	N.R.	8.0	[89]
Rice (40% sorbitol)	3.2	N.R.	23.0	
Cassava (30% glycerol)	1.7	38.8	11.0	[97]
Corn	1.2	22.7	62.6	[98]

N.R.: Not reported.

**Table 7:** Mechanical properties in biocomposites based on synthetic polymers derived from petroleum.

Biocomposite	Tensile Strength (Mpa)	Modulus of Elasticity (Mpa)	Tensile Strain (%)	Impact Resistance (Kj/M^2^)	Author
LDPE/NBF	18.6	645.0	22.3–40.3	12.3	[103]
LDPE/MBF1	26.9	889.3	N.R.	16.7	
LDPE/MBF2	29.2	912.6	N.R.	19.5	
Polyester/NPF	27.7	1038–1042	3.4–3.9	N.R.	[108]
Polyester/MPF	30.7	1229–1231	3.0–4.6	N.R.	
Epoxy/NBF	14.5	725	N.R.	2.2	[109]
Epoxy/MBF	33.6	1680	N.R.	12.2	

N.R.: Not reported. NBF: Native banana fibre; MBF: Modified banana fibre; NPF: Native plantain fibre; FPM: Modified plantain fibre.

**Table 8:** Tensile properties of TPS-based composites.

Biocomposite	Tensile Strength (Mpa)	Modulus Of Elasticity (Mpa)	Tensile Strain (%)	Author
TPS corn/MPF	21.6–29.2	3410–4010	1.7–2.3	[111]
TPS corn/NPF	3.87–4.23	88.2–106.1	12.06–12.4	[112]
TPS cassava/NBF	14.6	700	4.8	[113]
TPS cassava/MBF	24.8	3100	1.2	[113]

MPF: Modified plantain fibre; NPF: Native plantain fibre; NBF: Native banana fibre; MBF: Modified banana fibre.

**Table 9:** Mechanical properties of PHB-based biocomposites.

Biocomposite	Tensile Strength (Mpa)	Flexure Strength (Mpa)	Modulus of Elasticity At Flexure (Mpa)	Impact Resistance (Kj/M^2^)	Author
PHB	23.8–24.2	28.2–28.6	2655.2	8.1–8.5	[117]
PHB/MBF5%	25.9–27.1	34.9–35.3	2870.5	10.2–11	
PHB/MBF10%	19.9–21.1	30.2–30.6	2450.2	9.2–9.6	

MBF: Modified banana fibre.

**Table 10:** Mechanical properties of PLA-based biocomposites.

Biocomposite	Tensile Strength (MPa)	Modulus of Elasticity (Mpa)	Flexure Strength (MPa)	Impact Resistance (J/m)	Author
PLA/MBF1	78.6	7200	65.4	17.1	[119]
APLA/NPF	47–49	2575–2815	N.R.	N.R.	[120]
APLA/MPF2	50.3–52.7	2600–2820	N.R.	N.R.	
SPLA/NPF	46–51	2584–2796	N.R.	N.R.	
SPLA/MPF2	48.9–51.3	2600–2840	N.R.	N.R.	
PLA/NBF	13.2–16	4593–4669	N.R.	17.7–20.5	[118]
PLA/MBF3	14.5–17.5	4580–4692	N.R.	17.3–22.1	
PLA/NBF + C30B	42.3–57.7	4639–4827	N.R.	27.7–40.5	
PLA/MBF3 + C30B	44.7–57.3	5033–5141	N.R.	29.7–39.1	

N.R.: Not reported. MBF: Modified banana fibre; NPF: Native plantain fibre; NBF: Native banana fibre; MPF: Modified plantain fibre; APLA: Amorphous polylactic acid; SPLA: Semi-crystalline polylactic acid; PLA: Polylactic acid.

## Data Availability

Not applicable.
